# MASS-FIX for the detection of monoclonal proteins and light chain N-glycosylation in routine clinical practice: a cross-sectional study of 6315 patients

**DOI:** 10.1038/s41408-021-00444-0

**Published:** 2021-03-04

**Authors:** Patrick W. Mellors, Surendra Dasari, Mindy C. Kohlhagen, Taxiarchis Kourelis, Ronald S. Go, Eli Muchtar, Morie A. Gertz, Shaji K. Kumar, Francis. K. Buadi, Maria A. V. Willrich, John A. Lust, Prashant Kapoor, Martha Q. Lacy, David Dingli, Yi Hwa, Amie Fonder, Miriam Hobbs, Susan Hayman, Rahma Warsame, Nelson R. Leung, Yi Lin, Wilson Gonsalves, Mustaqeem Siddiqui, Robert A. Kyle, S. Vincent Rajkumar, David L. Murray, Angela Dispenzieri

**Affiliations:** 1 Department of Internal Medicine, Mayo Clinic, Rochester, MN USA; 2 Department of Laboratory Medicine and Pathology, Mayo Clinic, Rochester, MN USA; 3 Division of Hematology, Mayo Clinic, Rochester, MN USA

**Keywords:** Myeloma, Translational research

## Abstract

Immunoenrichment-based matrix assisted laser desorption ionization time-of-flight mass spectrometry (MALDI-TOF-MS), termed MASS-FIX, offers several advantages over immunofixation for the detection and isotyping of serum monoclonal protein, including superior sensitivity and specificity, the ability to differentiate therapeutic monoclonal antibodies, and the rapid identification of light chain (LC) N-glycosylation. We identified 6315 patients with MASS-FIX performed at our institution since 2018. Of these, 4118 patients (65%) with a wide array of plasma cell disorders (PCD), including rare monoclonal gammopathies of clinical significance, had a positive MASS-FIX. Two-hundred twenty-one (5%) of the MASS-FIX positive patients had evidence of LC N-glycosylation, which was more commonly identified in IgM heavy chain isotype, kappa LC isotype, and in diagnoses of immunoglobulin light chain (AL) amyloidosis and cold agglutinin disease (CAD) compared to other PCD. This cross-sectional study describes the largest cohort of patients to undergo MASS-FIX in routine clinical practice. Our findings demonstrate the widespread utility of this assay, and confirm that LC N-glycosylation should prompt suspicion for AL amyloidosis and CAD in the appropriate clinical context.

## Introduction

Since 2018, immunoenrichment-based matrix assisted laser desorption ionization time-of-flight mass spectrometry (MALDI-TOF-MS), termed MASS-FIX, has replaced serum immunofixation (sIFE) for the detection and isotyping of serum monoclonal protein (MP) at Mayo Clinic Rochester campus^1,2^. The advantages of MASS-FIX include its rapid throughput, high sensitivity and specificity for the detection of MP, and ability to differentiate therapeutic monoclonal antibodies^3,4^.

In addition, MASS-FIX can easily identify light chain (LC) N-glycosylation by its characteristic polytypic spectral pattern. LC N-glycosylation has diagnostic implications, as it is more common in immunoglobulin light chain (AL) amyloidosis and cold agglutinin disease (CAD) compared to other plasma cell disorders (PCD)^5,6^. In AL amyloidosis, LC N-glycosylation is present from the time of diagnosis of monoclonal gammopathy of undetermined significance (MGUS), and represents an independent risk factor for progression of MGUS to AL amyloidosis and other PCD^7,8^. Moreover, LC N-glycosylation has been implicated in the pathogenesis of amyloid fibril formation in AL amyloidosis^9^. However, the molecular mechanism has not been clarified.

The aim of this cross-sectional study is to describe the clinical utility of MASS-FIX for the detection of MP and LC N-glycosylation in routine clinical practice. Herein, we report our single institution experience with MASS-FIX in a cohort of 6315 patients.

## Methods

### Inclusion and exclusion criteria

MASS-FIX was performed on patient samples as previously described^1^. Demographics and laboratory data, including quantitative M-spike, serum free light chains (sFLC), and quantitative immunoglobulins at the time of MASS-FIX were recorded. For patients with multiple samples during the study period, only the initial MASS-FIX results were considered.

**Fig. 1:**
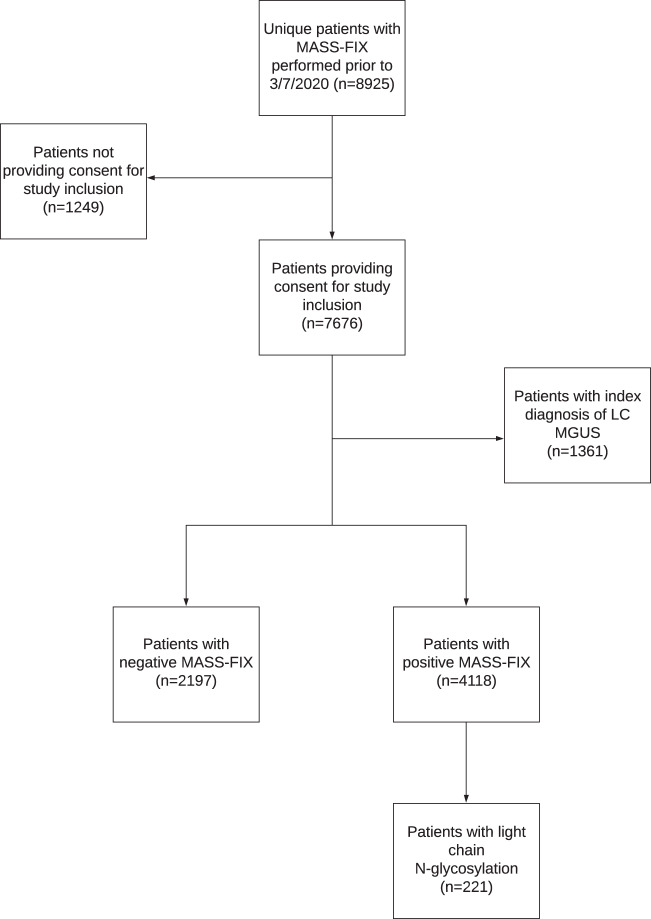
Consort diagram. Illustrating inclusion and exclusion criteria for 4118 MASS-FIX positive and 2197 MASS-FIX negative patients with MASS-FIX performed at Mayo Clinic from 7/24/2018 to 3/7/2020; LC MGUS light chain monoclonal gammopathy of undetermined significance.

Figure 1 illustrates the inclusion and exclusion criteria for the final cohort of 6315 patients with a positive (4118) and negative (2197) MASS-FIX. We initially identified 8925 patients with MASS-FIX performed between 7/24/2018 to 3/6/2020 and (1) a PCD diagnosis and/or (2) a diagnosis of non-AL amyloidosis (e.g., transthyretin amyloidosis (ATTR), AA amyloidosis, heavy chain (HC) amyloidosis, etc.). We included both treated and untreated patients at various stages of diagnosis and follow-up. Of these, 7676 provide consent for study enrollment.

For several reasons, we excluded patients if the only PCD diagnosis was LC MGUS (*n* = 1361). First, there are inconsistencies in the interpretation of diagnostic criteria for this disorder, and a diagnosis is often assigned inappropriately based on an abnormal FLC ratio alone, without evidence of an increase in the involved FLC^10,11^. Secondly, several institutions, including our own, have previously reported increases in the percentage of patients with elevated FLC ratios in routine clinical practice secondary to assay calibration drift^12,13^. Although FLC assay calibration at our institution was ultimately corrected by the manufacturer, patients tested between 2015 and 2017, particularly with marginal elevations in FLC ratio between 1.65 and 3.0, may have been inappropriately labeled as having LC MGUS.

Patients were considered to be MASS-FIX negative if (1) no MP was identified on MASS-FIX (*n* = 1069); (2) multiple nonspecific spectral peaks were identified consistent with immune reconstitution (*n* = 29); (3) the interpretation rendered was “cannot rule out MP” (*n* = 943); and (4) the only MP identified was consistent with a therapeutic monoclonal antibody (*n* = 156).

### LC N-glycosylation and AL amyloidosis evaluation

Spectra for 221 (5%) of the 4118 MASS-FIX positive patients had complex polytypic patterns in higher mass ranges consistent with LC N-glycosylation, as previously described^5^. Given the association between AL amyloidosis and LC N-glycosylation, medical records were reviewed to establish if patients with LC N-glycosylation were formally evaluated for AL amyloidosis by tissue biopsy. Criteria for the diagnosis of AL amyloidosis included the presence of a positive Congo Red stain that exhibited green birefringence under polarized light on tissue or bone marrow biopsy. AL subtype was identified by liquid chromatography/mass spectrometry (LC–MS). Patients were classified as having localized amyloidosis if the circulating MP was a different isotype than that identified on tissue biopsy, while those with concordant isotypes were categorized as having systemic AL amyloidosis. Patients were categorized as having amyloidosis of indeterminate type if tissue sampling was positive for Congo Red, but the sample was insufficient for subtyping, or if LC–MS could not identify a specific amyloid subtype.

### Statistical analysis

Pearson’s chi-squared test of independence and Fisher’s exact test were used to compare frequencies for categorical variables. The nonparametric Kruskal–Wallis test was used to compare medians for continuous variables. *P* values <0.01 were considered to be statistically significant. Statistical analyses were performed using JMP v14.1 software package (SAS Institute Inc., Cary, NC, USA).

## Results

### Demographic and laboratory characteristics

Demographic and laboratory characteristics for 4118 patients with MP detected by MASS-FIX stratified by presence (*n* = 221) and absence (*n* = 3897) of LC N-glycosylation are shown in Table 1. For MASS-FIX positive patients overall, 2476 were men (60%) and median age was 67 (interquartile range (IQR) 59–74). Median follow-up from diagnosis was 1.5 years (IQR 0.5–5.1) and median time from diagnosis of interest to MASS-FIX was 3.5 months (IQR 0.1–50.7). Of the 4118 MASS-FIX positive patients, 1299 (32%) were treated prior to MASS-FIX. There were no statistically significant differences in these parameters between non-LC N-glycosylated and LC N-glycosylated subgroups.

**Table 1:** Demographic and laboratory characteristics of 4118 MASS-FIX positive patients, stratified by non-LC N-glycosylated (3897) and LC N-glycosylated (221) subgroups.

	Total MASS-FIX Positive	No LC N-glycosylation	LC N-glycosylation	*P* value
Men	2476 (60)	2335 (60)	141 (64)	0.25
Age at diagnosis [median years (IQR)]	67 (59–74)	67 (59–74)	69 (59–74)	0.77
Follow-up from diagnosis [median years (IQR)]	1.5 (0.5–5.1)	1.5 (0.5–5.1)	1.6 (0.5–5.2)	0.57
Time from diagnosis to MASS-FIX [median months (IQR)]	3.5 (0.1–50.7)	3.3 (0.1–50.5)	5.0 (0.1–55.3)	0.09
Treated prior to MASS-FIX	1299 (32)	1221 (31)	78 (35)	0.22
Free light chains				
Increased FLC ratio (kappa)	1443 (39)	1333 (38)	110 (55)	**<0.001**
Decreased FLC ratio (lambda)	557 (15)	536 (15)	21 (10)	0.06
Normal FLC ratio	1681 (46)	1611 (46)	70 (35)	
Serum protein electrophoresis				
Quantifiable M-spike	1671 (41)	1538 (39)	133 (60)	0.18(78)
MASS-FIX characteristics				
IgG isotype^a^	2575 (63)	2436 (63)	139 (63)	0.91
IgA isotype^a^	703 (17)	685 (18)	18 (8)	**<0.001**
IgM isotype^a^	710 (17)	655 (17)	55 (25)	**0.002**
IgD/IgE isotype^b^	15 (<1)	15 (<1)	0	
Kappa light chain restricted^a^	2374 (58)	2201 (56)	173 (78)	**<0.001**
Lambda light chain restricted^a^	1886 (46)	1838 (47)	48 (22)	**<0.001**
Free heavy chain	2 (<1)	2 (<1)	0	
Light chain only	283 (7)	272 (7)	11 (5)	0.25
Kappa free light chain	121 (43)	115 (42)	6 (55)	
Lambda free light chain	162 (57)	157 (58)	5 (45)	
Monoclonal pattern	3626 (88)	3437 (88)	189 (85)	0.23
Biclonal pattern	228 (6)	210 (5)	18 (8)	0.08
Triclonal pattern	7 (<1)	4 (<1)	3 (1)	

Data are given as [*n* (%)] unless otherwise noted. IQR, interquartile range. FLC ratio was available for 3681 MASS-FIX positive patients, 3480 non-LC N-glycosylated patients, and 201 LC N-glycosylated patients. For the LC N-glycosylation subgroup, MASS-FIX categories for heavy chain isotypes, involved light chain, light chain only, and clonality are in reference to the glycosylated monoclonal protein.

Bolded *p* values indicate statistically significant differences between non-LC N-glycosylated and LC N-glycosylated groups.

^a^For the MASS-FIX positive group and non-LC N-glycosylated subgroup, light chain restriction and overall heavy chain isotype are not mutually exclusive due to biclonality; for the LC N-glycosylation subgroup, overall heavy chain isotype is mutually exclusive, with the exception of two patients with biclonal patterns and LC N-glycosylation of both clones (both patients were IgM kappa and IgG kappa).

^b^MASS-FIX is not set up to detect monoclonal IgD/IgE. Light chains detected by MASS-FIX and samples reflexed to standard, gel-based immunofixation.

Only 54% of MASS-FIX positive patients had an abnormal FLC ratio. For patients with and without LC N-glycosylation, 78% and 56% had a kappa restricted clone by MASS-FIX, respectively (*p* < 0.001). The overall percentage of patients with IgG HC isotype was identical for both groups (63%); however, IgA HC isotype was less common in the LC N-glycosylated subgroup than the non-LC N-glycosylated subgroup (8% versus 18%, respectively, *p* < 0.001), while IgM was more common (25% versus 17%, respectively, *p* = 0.002). Lastly, there were no significant differences in the percentages of patients with light chain only, monoclonal, and biclonal patterns.

### Diagnoses of patients with positive MASS-FIX on cross-sectional testing

**Fig. 2:**
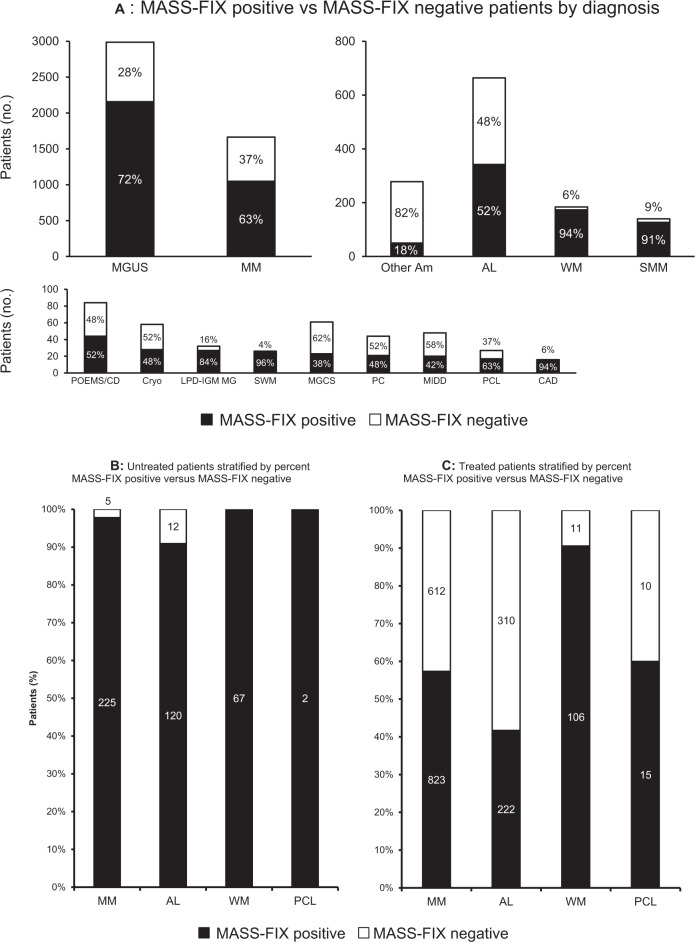
**A** MASS-FIX positive vs. MASS-FIX negative patients by diagnosis. MGUS monoclonal gammopathy of undetermined significance, MM multiple myeloma, Other Am other amyloidosis, AL immunoglobulin light chain amyloidosis, WM Waldenstrom’s macroglobulinemia, SMM smoldering multiple myeloma, POEMS/CD POEMS/Castleman’s disease, Cryo cryoglobulinemia, LPD-IGM MG lymphoproliferative disorder with monoclonal gammopathy, SWM smoldering Waldenstrom’s macroglobulinemia, MGCS monoclonal gammopathy of clinical significance, PC plasmacytoma, MIDD monoclonal immunoglobulin deposition disease, PCL plasma cell leukemia, CAD cold agglutinin disease. Note that MASS-FIX positive other amyloidosis has an associated MGUS. **B** Untreated patients stratified by percent MASS-FIX positive versus MASS-FIX negative. **C** Treated patients stratified by percent MASS-FIX positive versus MASS-FIX negative. MM multiple myeloma, AL immunoglobulin light chain amyloidosis, WM Waldenstrom’s macroglobulinemia, PCL plasma cell leukemia.

The distribution of MASS-FIX positive versus MASS-FIX negative patients by diagnosis is shown in Fig. 2A. MGUS, multiple myeloma (MM), and AL amyloidosis were the most common diagnoses evaluated with MASS-FIX, while Waldenstrom’s macroglobulinemia (WM) (94%), smoldering WM (SWM) (96%), smoldering multiple myeloma (SMM) (91%), lymphoproliferative disorder with monoclonal gammopathy (LPD-IGM MG) (84%), and CAD (94%) had the highest percentages of MASS-FIX positive patients. Specific diagnoses within the categories of other types of amyloidosis (“Other Am”) and monoclonal gammopathies of clinical significance (MGCS) are listed separately in Supplementary Tables 1, 2, respectively. Fifty patients (18%) with non-AL amyloidosis were MASS-FIX positive due to a concurrent MGUS. Patients with wild-type ATTR had a MGUS rate of 21%. A minority of patients with other potential MGCS diagnoses (*n* = 23, 38%) were MASS-FIX positive. Within this group, the most common diagnosis among MASS-FIX positive patients was membranoproliferative glomerulonephritis (*n* = 13, 57%). Supplementary Table 3 lists 25 patients with other amyloidosis types who had a concurrent PCD other than MGUS; in these cases, the PCD (MM, WM, etc.) was assigned as the primary diagnosis. Wild-type ATTR was the most common other amyloidosis type (52%), while MM was the most common PCD (52%).

**Fig. 3:**
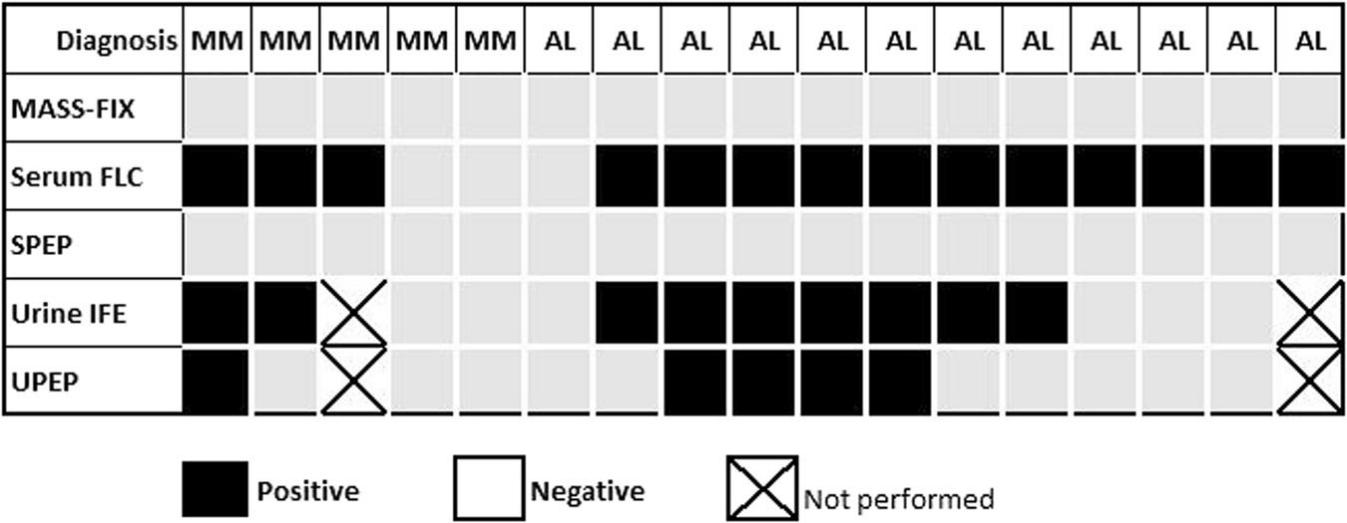
Serum and urine monoclonal protein study performance for untreated, MASS-FIX negative multiple myeloma and AL amyloidosis patients. Columns represent individual patients; FLC, free light chains; SPEP, serum protein electrophoresis; IFE, immunofixation; UPEP, urine protein electrophoresis.

Figures 2B and 2C stratify MASS-FIX results by treatment status for MM, AL amyloidosis, WM, and plasma cell leukemia (PCL). Figure 3 illustrates the results of serum and urine MP studies for five patients with untreated MM and 12 patients with untreated AL amyloidosis with negative MASS-FIX. Of the five MM patients, two had nonsecretory MM and three had LC MM. The two patients with nonsecretory MM had no MP detected by a combination of SPEP, MASS-FIX, sFLC, urine protein electrophoresis (UPE), and urine IFE (uIFE). Of the three patients with LC MM, all had elevations in involved FLC and an abnormal FLC ratio. With respect to urine studies, one patient had neither UPE nor uIFE performed, one had a quantifiable M-spike on UPE and a positive uIFE, and one had negative UPE with a positive uIFE.

Twelve patients (9%) with untreated AL amyloidosis had a negative MASS-FIX. All 12 were evaluated with sFLC assay and had an elevated involved FLC, and all but one patient had an abnormal sFLC ratio. Eleven patients (92%) were evaluated with both uIFE and UPE; of these, eight patients (73%) had a positive uIFE, and four of these were also positive by UPE (two had a quantifiable M-spikes and two had small, nonquantifiable abnormalities). Ultimately, sFLC captured three additional AL amyloidosis diagnoses that would have been missed with a combination of MASS-FIX, SPEP, UPE, and uIFE. An additional AL amyloidosis diagnosis was captured with sFLC in a patient with negative MASS-FIX and SPEP in whom urine studies were not performed. All untreated WM and PCL patients were MASS-FIX positive.

### Diagnoses of patients with LC N-glycosylation on cross-sectional testing

**Fig. 4:**
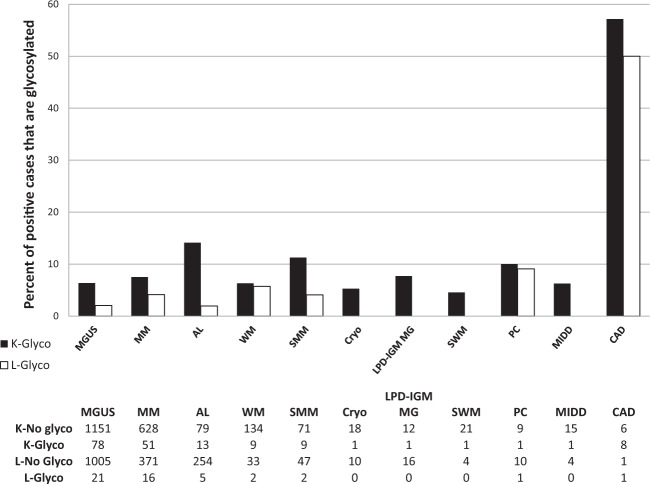
Glycosylation status by diagnosis and light chain restriction. MGUS monoclonal gammopathy of undetermined significance, MM multiple myeloma, AL immunoglobulin light chain amyloidosis, WM Waldenstrom’s macroglobulinemia, SMM smoldering multiple myeloma, Cryo cryoglobulinemia, LPD-IGM MG lymphoproliferative disorder with monoclonal gammopathy, SWM smoldering Waldenstrom’s macroglobulinemia, PC plasmacytoma, MIDD monoclonal immunoglobulin deposition disease, and CAD cold agglutinin disease. Note that non-glycosylated kappa and lambda light chains (LC) are not mutually exclusive due to bi and triclonality. However, glycosylated LC are mutually exclusive, as LC isotype is in reference to the glycosylated LC, and no patients had glycosylated LC of different isotypes.

Figure 4 illustrates the percentage of patients with LC N-glycosylation by PCD diagnosis, stratified by LC isotype. For all PCD, kappa LC N-glycosylation was more common than lambda LC N-glycosylation (78% versus 22%, respectively). CAD had the highest percentage of LC N-glycosylation for both kappa (eight patients, 57%) and lambda LC (one patient, 50%). For AL amyloidosis, 13 patients (14.1%) with kappa LC restriction and five patients (1.9%) with lambda LC restriction had LC N-glycosylation. Results were similar the subgroup of AL amyloidosis patients who were untreated (16.7% of kappa LC and 2.2% lambda LC). Of note, LC N-glycosylation was not identified in patients with POEMS syndrome, PCL, or other MGCS diagnoses.

Of the 221 patients with LC N-glycosylation, 105 (48%) were evaluated for AL amyloidosis with various combinations of fat aspirate (FA), bone morrow biopsy (BMB), and tissue biopsy stained for Congo Red with reflex amyloid subtyping by LC–MS, as outlined in Table 2. Patients with a final diagnosis of SMM had the highest rate of inquiry for amyloidosis (64%), excluding those patients who were ultimately diagnosed with AL amyloidosis; only 38% of MGUS and 53% of MM patients had any tissue biopsy looking for AL amyloidosis. With respect to biopsy site, patients with AL amyloidosis (88%) and MM (82%) were the most likely to be evaluated with BMB, while patients with MGUS were most likely to be evaluated with FA (74%).

**Table 2:** Patients with LC N-glycosylation evaluated for AL amyloidosis by diagnosis with biopsy site and indication.

	AL	MGUS	SMM	MM	WM
Number with LC N-glycosylation	18	99	11	67	11
Evaluated for AL amyloidosis	18 (100)	38 (38)	7 (64)	36 (53)	6 (55)
Biopsy site^a^					
Fat aspirate	4 (22)	28 (74)	4 (57)	16 (48)	4 (67)
Bone marrow	16 (88)	17 (45)	5 (71)	27 (82)	5 (67)
Endomyocardial	1 (6)	2 (5)		1 (3)	
Renal	6 (33)	2 (5)	1 (14)	1 (3)	1 (17)
Nerve		2 (5)			
Other	4 (22)	3 (8)		2 (6)	
Indication for biopsy					
Cardiac sx^b^	4 (22)	8 (21)	1 (14)	6 (17)	
Renal sx^c^	6 (33)	4 (11)		2 (6)	1 (17)
Neuropathic sx^d^	4 (22)	15 (39)	2 (29)	5 (14)	1 (17)
Other sx^e^	4 (22)	11 (39)	4 (57)	23 (64)	4 (67)

Data are given as [*n* (%)]; percentages are in reference to column/diagnosis. Patients with the following diagnoses and LC N-glycosylation were not evaluated for AL amyloidosis: cold agglutinin disease, cryoglobulinemia, lymphoproliferative disorder with monoclonal gammopathy, smoldering Waldenstrom’s macroglobulinemia, plasmacytoma, and monoclonal immunoglobulin deposition disease.

*AL* AL amyloidosis, *MGUS* monoclonal gammopathy of undetermined significance, *SMM* smoldering multiple myeloma, *MM* multiple myeloma, *WM* Waldenstrom’s macroglobulinemia, *Sx* symptoms or signs.

^a^Biopsy sites are not mutually exclusive, as multiple biopsy types could be obtained in the same patient.

^b^Cardiac indications for biopsy include unexplained dyspnea, heart failure, atrial fibrillation, and abnormal transthoracic echocardiogram.

^c^Renal indications for biopsy include unexplained proteinuria, acute kidney injury, or chronic kidney disease

^d^Neuropathy indications for biopsy include peripheral or autonomic neuropathy.

^e^Other indications include unknown, vision loss, rash, myalgia, macroglossia, arthralgia, hoarseness, diarrhea, dyspepsia, gastrointestinal bleeding, coagulopathy, fatigue.

Of the 105 patients who were evaluated for amyloidosis, 18 (17%) were diagnosed as AL amyloidosis. One of these patients with AL amyloidosis had a concurrent diagnosis of familial ATTR by BMB. Three additional patients, who were all diagnosed with MM, had a positive Congo Red on BMB; one was diagnosed with wild-type ATTR, and two had amyloid of indeterminate type. Therefore, of the 75 patients with other types of amyloidosis (50 with an associated MGUS and 25 with a non-MGUS PCD), four patients (5.3%) had LC N-glycosylation. For the remaining patients who were evaluated for amyloidosis, one had an equivocal Congo Red stain, and 83 patients (79%) had no evidence of amyloid deposition in biopsied tissue.

As outlined in Table 2, excluding “other” indications, unexplained renal disease was the most common clinical indication for biopsy among patients with an AL amyloidosis diagnosis (6, 33%), while neuropathy was the most common indication for MGUS and SMM (15, 39% and 2, 29%, respectively). Cardiac symptomatology was the most common indication in MM (6, 17%). One patient with WM had a renal indication for biopsy, and one had a neuropathy indication.

## Discussion

This cross-sectional study describes the largest cohort of patients to date to be evaluated for serum MP using a high throughput, mass spectrometry-based assay in a routine clinical setting. Our data demonstrate that MASS-FIX detects MPs in a wide variety of PCD, including rare disorders such as AL amyloidosis and MGCS. Moreover, HC isotype, LC restriction, clonality, and LC N-glycosylation are easily characterized, as are therapeutic monoclonal antibodies (data not shown).

While this cross-sectional study was not specifically designed to evaluate the sensitivity and specificity of MASS-FIX for the detection of MP, we note that among previously untreated patients, only 5 with MM (2%) and 12 with AL amyloidosis (9%) had a negative MASS-FIX. Considering serum studies alone, the addition of sFLC to MASS-FIX captured three additional MM diagnoses and 12 additional AL amyloidosis diagnoses, improving sensitivity from 91 to 100% for the latter. This is consistent with previous results from our group, in which the addition of sFLC to serum MASS-FIX improved diagnostic sensitivity for untreated AL amyloidosis patients from 80 to 100%, and is comparable to sIFE, in which sensitivity increases from 74 to 97% with the addition of sFLC^3,14^. This highlights a potential limitation of serum MASS-FIX without an accompanying sFLC in detecting MP in such patients in routine clinical practice.

We confirm the findings of previous studies demonstrating high rates of LC N-glycosylation in CAD and AL amyloidosis^5,6,8^. We expand upon previous work in identifying additional PCD with LC N-glycosylation, including cryoglobulinemia, plasmacytoma, and monoclonal immunoglobulin deposition disease. Similar to our prior report, we identified LC N-glycosylation in 5% of patients with other types of amyloidosis and a concurrent plasma cell disorder^3^. Although the numbers are relatively small, none of the patients with diagnoses of PCL, POEMS/Castleman’s disease, scleromyxdema, Schnitzler’s syndrome, or necrobiotic xanthogranuloma had evidence of N-glycosylated LCs.

Similar to previous reports, LC N-glycosylation was more commonly identified in kappa than in lambda LC in AL amyloidosis and for PCD in general. Consistent with these results, bottom up proteomic analyses have demonstrated an association between KV1 gene family usage within the kappa LC variable region and LC N-glycosylation^5,15^. However, the percentage of kappa and lambda LC with N-glycosylation in patients with previously untreated AL amyloidosis was lower than previously reported (kappa: 16.7% versus 32.8%; lambda 2.2% versus 10.2%)^5^. The smaller sample size in our study could be responsible for these observed differences.

Of note, approximately one-half of all patients with LC N-glycosylation were formally evaluated for AL amyloidosis with BMB and/or tissue biopsy. Of those who were evaluated, approximately half had a FA and two thirds had a BMB performed, which have individual sensitivities of 70–75% and combined sensitivities of 85–90% for the diagnosis of AL amyloidosis^16^. Follow-up on these patients is short, but prior studies have shown that patients with MGUS and N-glycosylation have a sixfold higher rate of progression to a malignant PCD than their non-glycosylated counterparts^8^.

In conclusion, MASS-FIX can identify MP and LC N-glycosylation in a wide range of PCD. The presence of LC N-glycosylation can serve as an important diagnostic clue and should raise suspicion for the presence of AL amyloidosis or CAD in the appropriate clinical context and should be a harbinger of increased risk of progression. Finally, it may play a pathogenic role in the development of amyloid fibrils, making LC-bound carbohydrate moieties an intriguing therapeutic target.

## Supplementary information

Supplemental Table 1

Supplemental Table 2

Supplemental Table 3
