# Correction: MASS-FIX for the detection of monoclonal proteins and light chain N-glycosylation in routine clinical practice: a cross-sectional study of 6315 patients

**DOI:** 10.1038/s41408-021-00496-2

**Published:** 2021-06-07

**Authors:** Patrick W. Mellors, Surendra Dasari, Mindy C. Kohlhagen, Taxiarchis Kourelis, Ronald S. Go, Eli Muchtar, Morie A. Gertz, Shaji K. Kumar, Francis. K. Buadi, Maria A. V. Willrich, John A. Lust, Prashant Kapoor, Martha Q. Lacy, David Dingli, Yi Hwa, Amie Fonder, Miriam Hobbs, Susan Hayman, Rahma Warsame, Nelson R. Leung, Yi Lin, Wilson Gonsalves, Mustaqeem Siddiqui, Robert A. Kyle, S. Vincent Rajkumar, David L. Murray, Angela Dispenzieri

**Affiliations:** 1 Department of Internal Medicine, Mayo Clinic, Rochester, MN USA; 2 Department of Laboratory Medicine and Pathology, Mayo Clinic, Rochester, MN USA; 3 Division of Hematology, Mayo Clinic, Rochester, MN USA

**Keywords:** Myeloma, Translational research

Correction to: *Blood Cancer Journal*

10.1038/s41408-021-00444-0 published online 4 March 2021

Table 1 in this article was published with some comments in the original version. We apologize for the mistake. This has been corrected below:

**Table 1:** Demographic and laboratory characteristics of 4118 MASS-FIX positive patients, stratified by non-LC N-glycosylated (3897) and LC N-glycosylated (221) subgroups.

	Total MASS-FIX Positive	No LC N-glycosylation	LC N-glycosylation	*P* value
Men	2476 (60)	2335 (60)	141 (64)	0.25
Age at diagnosis [median years (IQR)]	67 (59–74)	67 (59–74)	69 (59–74)	0.77
Follow-up from diagnosis [median years (IQR)]	1.5 (0.5–5.1)	1.5 (0.5–5.1)	1.6 (0.5–5.2)	0.57
Time from diagnosis to MASS-FIX [median months (IQR)]	3.5 (0.1–50.7)	3.3 (0.1–50.5)	5.0 (0.1–55.3)	0.09
Treated prior to MASS-FIX	1299 (32)	1221 (31)	78 (35)	0.22
Free light chains				
Increased FLC ratio (kappa)	1443 (39)	1333 (38)	110 (55)	**<0.001**
Decreased FLC ratio (lambda)	557 (15)	536 (15)	21 (10)	0.06
Normal FLC ratio	1681 (46)	1611 (46)	70 (35)	
Serum protein electrophoresis				
Quantifiable M-spike	1671 (41)	1538 (39)	133 (60)	0.18(78)
MASS-FIX characteristics				
IgG isotype^a^	2575 (63)	2436 (63)	139 (63)	0.91
IgA isotype^a^	703 (17)	685 (18)	18 (8)	**<0.001**
IgM isotype^a^	710 (17)	655 (17)	55 (25)	**0.002**
IgD/IgE isotype^b^	15 (<1)	15 (<1)	0	
Kappa light chain restricted^a^	2374 (58)	2201 (56)	173 (78)	**<0.001**
Lambda light chain restricted^a^	1886 (46)	1838 (47)	48 (22)	**<0.001**
Free heavy chain	2 (<1)	2 (<1)	0	
Light chain only	283 (7)	272 (7)	11 (5)	0.25
Kappa free light chain	121 (43)	115 (42)	6 (55)	
Lambda free light chain	162 (57)	157 (58)	5 (45)	
Monoclonal pattern	3626 (88)	3437 (88)	189 (85)	0.23
Biclonal pattern	228 (6)	210 (5)	18 (8)	0.08
Triclonal pattern	7 (<1)	4 (<1)	3 (1)	

Data are given as [*n* (%)] unless otherwise noted. IQR, interquartile range. FLC ratio was available for 3681 MASS-FIX positive patients, 3480 non-LC N-glycosylated patients, and 201 LC N-glycosylated patients. For the LC N-glycosylation subgroup, MASS-FIX categories for heavy chain isotypes, involved light chain, light chain only, and clonality are in reference to the glycosylated monoclonal protein.

Bolded *p* values indicate statistically significant differences between non-LC N-glycosylated and LC N-glycosylated groups.

^a^For the MASS-FIX positive group and non-LC N-glycosylated subgroup, light chain restriction and overall heavy chain isotype are not mutually exclusive due to biclonality; for the LC N-glycosylation subgroup, overall heavy chain isotype is mutually exclusive, with the exception of two patients with biclonal patterns and LC N-glycosylation of both clones (both patients were IgM kappa and IgG kappa).

^b^MASS-FIX is not set up to detect monoclonal IgD/IgE. Light chains detected by MASS-FIX and samples reflexed to standard, gel-based immunofixation.

The original article has been updated.

