# Supplementary material for: MASS-FIX for the detection of monoclonal proteins and light chain N-glycosylation in routine clinical practice: a cross-sectional study of 6315 patients
Source: Blood Cancer J. 2021 Mar 4;11(3):50. doi: 10.1038/s41408-021-00444-0 (PMC7933343; doi:10.1038/s41408-021-00444-0)
Supplement: Supplementary file 1 — Supplemental Table 1 [file 41408_2021_444_MOESM1_ESM.docx]

| **Supplemental Table 1:** Diagnoses for 278 patients with other types of amyloidosis (“Other Am”) and concurrent MGUS | | |
| --- | --- | --- |
|  | **MASS-FIX Positive** | **MASS-FIX Negative** |
| Wild type ATTR | 35 (21) | 129 (79) |
| AA amyloidosis | 4 (67) | 2 (33) |
| Hereditary ATTR | 4 (17) | 19 (83) |
| Localized AL^*^ | 3 (5) | 59 (95) |
| Amyloidosis, indeterminate type | 3 (25) | 9 (75) |
| Heavy chain amyloidosis | 1 (25) | 3 (75) |
| Other amyloid subtype |  | 7 (100) |
| **Total** | 50 (18) | 228 (82) |
| Data are given as [n (%)]; percentages refer to MASS-FIX positive versus MASS-FIX negative for each diagnosis  ATTR, transthyretin amyloidosis  ^*^Patients with a circulating monoclonal protein of a different isotype than that isolated from amyloidoma by tissue biopsy | | |
