# Supplementary material for: MASS-FIX for the detection of monoclonal proteins and light chain N-glycosylation in routine clinical practice: a cross-sectional study of 6315 patients
Source: Blood Cancer J. 2021 Mar 4;11(3):50. doi: 10.1038/s41408-021-00444-0 (PMC7933343; doi:10.1038/s41408-021-00444-0)
Supplement: Supplementary file 2 — Supplemental Table 2 [file 41408_2021_444_MOESM2_ESM.docx]

| **Supplemental Table 2:** Diagnoses for 61 patients with potential monoclonal gammopathies of clinical significance (MGCS) | | |
| --- | --- | --- |
|  | **MASS-FIX Positive** | **MASS-FIX Negative** |
| Membranoproliferative glomerulonephritis | 13 (32) | 28 (68) |
| Capillary leak syndrome | 3 (60) | 2 (40) |
| Scleromyxedema | 2 (100) |  |
| Schnitzler’s syndrome | 2 (67) | 1 (33) |
| Necrobiotic xanthogranuloma | 2 (100) |  |
| Acquired Fanconi syndrome | 1 (100) |  |
| Heavy chain deposition disease with or without light chain deposition disease |  | 4 (100) |
| Membranous nephropathy |  | 1 (100) |
| Fibrillary glomerulonephritis |  | 1 (100) |
| Tempi Syndrome |  | 1 (100) |
| **Total** | 23 (38) | 38 (62) |
| Data are given as [n (%)]; percentages refer to MASS-FIX positive versus MASS-FIX negative for each diagnosis | | |
