# Supplementary material for: MASS-FIX for the detection of monoclonal proteins and light chain N-glycosylation in routine clinical practice: a cross-sectional study of 6315 patients
Source: Blood Cancer J. 2021 Mar 4;11(3):50. doi: 10.1038/s41408-021-00444-0 (PMC7933343; doi:10.1038/s41408-021-00444-0)
Supplement: Supplementary file 3 — Supplemental Table 3 [file 41408_2021_444_MOESM3_ESM.docx]

| **Supplemental Table 3:** Diagnoses for 25 MASS-FIX positive patients with plasma cell disorders and concurrent other types of amyloidosis | | | |
| --- | --- | --- | --- |
| **Other Amyloidosis Type** |  | **Plasma Cell Disorder** |  |
| Wild type ATTR | 13 (52) | MM | 13 (52) |
| Amyloidosis, indeterminate type | 9 (36) | AL amyloidosis | 4 (16) |
| AA amyloidosis | 2 (8) | SMM | 3 (12) |
| Apolipoprotein A-IV amyloidosis | 1 (4) | WM | 2 (8) |
|  |  | Other MGCS | 2 (8) |
|  |  | SWM | 1 (4) |
| Data are given as [n (%)]; percentages are in reference to columns  ATTR, transthyretin amyloidosis; MM, multiple myeloma; SMM, smoldering multiple myeloma; WM, Waldenstrom’s macroglobulinemia; SWM, smoldering Waldenstrom’s macroglobulinemia; MGCS, monoclonal gammopathy of clinical significance | | | |
